# Firemaster^®^ 550 and its components isopropylated triphenyl phosphate and triphenyl phosphate enhance adipogenesis and transcriptional activity of peroxisome proliferator activated receptor (Pparγ) on the adipocyte protein 2 (aP2) promoter

**DOI:** 10.1371/journal.pone.0175855

**Published:** 2017-04-24

**Authors:** Emily W. Y. Tung, Shaimaa Ahmed, Vian Peshdary, Ella Atlas

**Affiliations:** Environmental Health Science and Research Bureau, Health Canada, Ottawa, Ontario, Canada; INIA, SPAIN

## Abstract

Firemaster^®^ 550 (FM550) is a chemical mixture currently used as an additive flame retardant in commercial products, and is comprised of 2-ethylhexyl-2,3,4,5-tertrabromobenzoate (TBB), bis(2-ethylhexyl) tetrabromophthalate (TBPH), triphenyl phosphate (TPP), and isopropylated triphenyl phosphate (IPTP). Animal and *in vitro* studies suggest that FM550, TPP and IPTP may have adipogenic effects and may exert these effects through PPARγ activation. Using murine 3T3-L1 preadipocytes, we investigated the detailed expression of transcription factors and adipogenic markers in response to FM550 and its components. Further we investigated the mechanism of action of the peroxisome proliferator-activated receptor gamma (PPAR**γ**) on downstream targets of the receptor by focussing on the mature adipocyte marker, adipocyte protein 2 (aP2). In addition, we set to elucidate the components responsible for the adipogenic effects seen in the FM550 mixture. We show that FM550 and its components TPP, IPTP, and TBPH, but not TBB induced lipid accumulation in a dose-dependent manner. Interestingly, despite displaying enhanced lipid accumulation, TBPH did not alter the mRNA or protein expression of terminal differentiation markers. In contrast, FM550, TPP, and IPTP treatment enhanced lipid accumulation, and mRNA and protein expression of terminal differentiation markers. To further delineate the mechanisms of action of FM550 and its components we focussed on *aP2* promoter activity. For this purpose we used the enhancer region of the mouse *aP2* promoter using a 584-bp reporter construct containing an active PPRE located 5.4 kb away from the transcription start site of aP2. Exposure to FM550, IPTP, and TPP significantly increased PPARγ mediated *aP2* enhancer activity. Furthermore, we show that TPP- and IPTP-dependent upregulation of aP2 was significantly inhibited by the selective PPAR*γ* antagonist GW9662. In addition, chromatin immunoprecipitation experiments showed that IPTP and TPP treatment led to the recruitment of PPARγ to the regulatory region of *aP2*.

## Introduction

Environmental chemicals found in consumer products can act as endocrine disrupting compounds (EDCs), which are defined as compounds that can interfere with the endocrine system. Some of these compounds have been also identified as potential obesogens, compounds that contribute to adipogenesis and to the development of obesity, in a variety of *in vivo* and *in vitro* systems [1–4]. The 3T3-L1 system is particularly well-studied and characterized, allowing for the elucidation of mechanisms of action of chemicals and identification of affected metabolic pathways in response to environmental pollutants [2,5,6]. The 3T3-L1 cells are preadipocytes, committed to the adipose lineage that will differentiate following exposure to a differentiation cocktail consisting of a cAMP enhancer, insulin, and either a glucocorticoid receptor or a PPARγ agonist [7]. The differentiation from preadipocyte to mature adipocyte is a tightly coordinated process, where the temporal expression of transcription factors is required to induce morphological changes, ultimately resulting in the formation of a lipid-accumulating mature adipocyte [7,8]. Transcriptional regulation of preadipocyte differentiation in this model system is well-characterized with the identification of key mediators including CCAAT/enhancer binding proteins (C/EBP) and PPARγ [9]. Following exposure to the classical differentiation cocktail, C/EBPβ and C/EBPδ are expressed within 4 hours of induction, and are responsible for initiating transcription of C/EBPα and PPARγ which are required to coordinate the expression of genes required for mature adipocyte formation [10,11]. Both C/EBPα and PPARγ expression are involved in the expression of known markers of adipogenesis, including aP2 [8].

aP2 is a cytosolic protein involved in lipid transport, acting as a fatty acid chaperone protein that regulates lipid trafficking and signaling in the mature adipocyte [12]. The aP2 promoter contains several PPAR response elements (PPREs) approximately 5.5kb upstream from the transcriptional start site, and is therefore directly regulated in part by PPARγ [13]. Functionally, aP2 has been correlated with obesity, insulin resistance, and atherosclerosis in murine models [14]. In humans, a genetic variant of the aP2 gene that results in reduced levels of aP2 was correlated with a lowered risk of type 2 diabetes and coronary artery disease when compared to the wild-type [15]. Studies suggest that aP2 has a role in metabolic disorders, and therefore its transcriptional regulation by environmental pollutants, such as Firemaster^®^ 550 (FM550) components, through nuclear receptors has important functional implications on the onset of obesity and metabolic syndrome.

Of the novel EDCs being identified, preliminary findings have shown that the proprietary flame retardant mixture FM550 exhibits PPARγ activation capability. FM550 is a proprietary mixture consisting of brominated and aryl phosphate ester compounds. The mixture is comprised of the following chemicals: bis (2-ethylhexyl) tetrabromophthalate (TBPH) (8%), 2-ethylhexyl-2,3,4,5-tetrabromobenzoate (TBB) (30%), triphenyl phosphate (TPP) (17%) and isopropylated triphenyl phosphates (IPTP) (45%) [16,17]. Exposure to these chemicals is likely wide spread as their use is not limited only to their flame retardant capabilities. For example, the FM550 component TPP is also used as a high-production volume plasticizer [18]. Similarly, TBPH is utilized as a plasticizer in polyvinyl chloride and neoprene rubber [19]. IPTP can also be found in hydraulic fluids, lubricants and lubricant additives [20]. Furthermore, studies have shown that these components may leach from the products to which they were added. Their widespread detection in both indoor and outdoor environments suggests that human exposure may be ubiquitous, and may impact human health [21–23].

The purpose of our study was to determine FM550’s and its components’ adipogenic potential, using 3T3-L1 preadipocytes, and to determine their ability to mediate PPARγ activation on the endogenous aP2 regulatory region. We determined by lipid accumulation that FM550 induced adipogenesis, and then deduced which components were responsible for the mixture’s effect. Further, we evaluated the mode of action by examining the temporal expression levels of key transcription factors throughout the differentiation process. In addition, we show an increase in the expression levels of terminal differentiation markers after treatment with both FM550 and its individual components. Further, we were able to show the upregulation and subsequent recruitment of PPARγ to the endogenous *aP2* regulatory region in response to FM550 components.

## Materials and methods

### Reagents

DMEM/low glucose media was purchase from HyClone Laboratories (Thermo Scientific, Logan, Utah, U.S.A.). Cell maintenance media was composed of DMEM/low glucose media and 10% bovine calf serum (ATCC, Manassas, Virginia, U.S.A.). Cell differentiation media consisted of DMEM/low glucose media, 10% fetal bovine serum (ATCC, Manassas, Virginia, U.S.A.) and 1% penicillin/streptomycin (Wisent Inc., Saint-Bruno, Quebec, Canada). Chemicals were purchased as follows: human insulin (Roche Diagnostics, Indianapolis, Indiana, U.S.A.); 3-isobutyl-1-methylxanthine (IBMX), dexamethasone (DEX), troglitazone (TROG), triphenyl phosphate (TPP), diphenyl phosphate (DPP), and dimethyl sulfoxide (DMSO) (Sigma-Aldrich, Oakville, Ontario, Canada); 2-ethylhexyl-2,3,4,5-tetrabromobenzoate (TBB) and bis(2-ethylhexyl) tetrabromophthalate (Toronto Research Chemicals, Toronto, Ontario, Canada). Isopropylated triphenyl phosphate (IPTP) was a generous gift from Warren Casey (NIEHS, US). Firemaster^®^ 550 was a generous gift from Brock Chittam (Wellington Laboratories, Canada). The PPARγ inhibitor GW9662 was purchased from Sigma Aldrich (M6191). Antibodies were purchased from the following manufacturers: fatty acid binding protein 4 (Fabp4 also known as aP2) and lipoprotein lipase (LPL) (AF3150 and AF7197, R&D Systems, Minneapolis, Minnesota, U.S.A); perilipin (PLIN), peroxisome proliferator-activated receptor γ (PPARγ), β-actin (9349, 4970, 2443, Cell Signaling Technology, Danvers, Massachusetts, U.S.A).

### Differentiation of 3T3-L1 preadipocytes into mature adipocytes

3T3-L1 preadipocytes (ATCC^®^ CL-173^™^) purchased from the American Type Culture Collection were differentiated as previously described [2]. Briefly, 3T3-L1 cells were seeded in maintenance media, allowed to reach confluence, and maintained for an additional 2 days after confluence prior to treatment with the differentiation cocktail. The differentiation cocktail was prepared in differentiation media and consisted of 500 μM of the cAMP enhancer IBMX (M), 100 nM of human insulin (I), and the test chemical (0–200 μM Firemaster^®^ 550, 0–10 μM IPTP, 0–20 μM TBB, 0–20 μM TPP, 0–20 μM TBPH, or 0–20 μM DPP, the main TPP metabolite) or positive control [250 nM dexamethasone (MID) or 5 μM troglizatone (MIT)]. Negative controls will hereby be indicated by MI (denoted 0). After 2 days of incubation, media was changed to contain 100 nM insulin and the test chemical. For the positive controls, cells that had received MID were replenished with media containing only insulin after day 2. Cells that had received MIT were replenished with insulin and troglitazone at day 2 and day 4. A final media change was made 4 days after the initiation of the differentiation process. For the PPARγ antagonist study, 5 μM of the irreversible antagonist GW9662 (Sigma Aldrich) was added to the differentiation media and replaced twice a day due to its short half-life [5]. Cells were then collected at various time points for analyses.

### Nile red staining of lipids

To assess the extent of lipid accumulation after treatment with test chemicals, preadipocytes were seeded on to black, clear-bottomed 96 well plates coated with collagen and exposed to the various chemicals indicated above. Following 9 days of treatment, cells were fixed with 4% paraformaldehyde and stained with Nile red (1 μg/mL) to identify lipid droplets and DAPI (1 μg/mL) to stain nuclei. Nile red fluorescence was measured at 485/528 nm (excitation/emission) and DAPI at 360/460 nm (excitation/emission) using a fluorescent plate reader (Synergy 2 Microplate Reader, BioTek Instruments Inc., Winooski, VT, U.S.A.). Nile red values were normalized to DAPI values and are relative to MI control. Cells were imaged at 200x magnification on a fluorescent microscope (Olympus IX71).

### PPARγantagonist studies for lipid accumulation

3T3-L1 preadipocytes were differentiated as described above. At days 2, 4, and 6 of differentiation media was changed to contain 100 nM insulin and the test chemical. For the positive controls, cells that had received MID were replenished with media containing only insulin. Cells that had received MIT were replenished with insulin and troglitazone at indicated time points. For addition of the PPARγ antagonist study, 5 μM of the irreversible antagonist GW9662 (Sigma Aldrich) or vehicle control were added twice a day due to its short half-life [5]. Lipid accumulation was assessed as described above.

### Western blotting for terminal differentiation markers

To measure the protein expression of terminal differentiation markers, 3T3-L1 preadipocytes were seeded on to 6-well dishes and treated with the aforementioned differentiation protocol. On day 9 of treatment, cells were collected in RIPA buffer (20 mM Tris pH 7.5, 150 mM NaCl, 1 mM EDTA, 1% sodium deoxycholate, 2% NP-40, 0.4% SDS, 10% glycine) containing protease inhibitors (Roche Diagnostics, Indianapolis, Indiana, U.S.A.) and sonicated to lyse cells. 20 μg of protein were separated on 15% polyacrylamide gels and proteins transferred to PVDF membranes. Membranes were probed with antibodies against aP2 (1:500), LPL (1:1000), PLIN (1:1000), or β-actin (1:1000) followed by appropriate HRP-linked secondary antibodies. Blots were subsequently developed using Clarity Western ECL Substrate (BioRad, Hercules, California, U.S.A.). Relative optical densities were quantified using Image Lab software (BioRad), and values for all terminal differentiation markers were normalized to β-actin levels.

### mRNA expression of transcription factors and adipocyte markers

To assess the expression of transcription factors and markers important for adipocyte differentiation, cells were harvested on days 2, 4, 6, and 9 or day 6 for the PPARγ antagonist study. Total RNA was isolated using the RNeasy Mini kit (Qiagen, Mississauga, Canada), followed by reverse transcription using iScript cDNA Synthesis Kit (BioRad). Expression profiles of the following genes were generated using SsoFast EvaGreen supermix (BioRad): *aP2*, *Lpl*, *Plin*, C/CAAT-enhancer binding protein α (*C/ebpα*), and *Pparγ*. Primers used are as follows: adipocyte protein 2 (aP2) forward 5`-GGAAGCTTGTCTCCAGTGAA-3` and reverse 5`-GCGGTGATTTCATCGAATTC-3`; peroxisome proliferator-activated receptor gamma (Pparγ) 5`-GCCTGCGGAAGCCCTTTGGT-3` and reverse 5`-GCAGTTCCAGGGCCTGCAGC-3`; perilipin (Plin) forward 5`-TTGGGGATGGCCAAAGAGAC-3` and reverse 5`-CTCACAAGGCTTGGTTTGGC-3`; lipoprotein lipase (Lpl) 5`- CAGGATGTGGCCCGGTTTAT-3` and reverse 5`- CGGGGCTTCTGCATACTCAA-3`; and CCAAT/enhancer-binding protein alpha (Cepbα) forward 5`- TGCGCAAGAGCCGAGATAAA-3` and reverse 5`- CCTTGACCAAGGAGCTCTCA-3`. β-actin: Forward- GACTTCGAGCAAGAGATGGC, Reverse- CCAGACAGCACTGTGTTGGC. All genes were amplified using BioRad SsoFast SYBR Green 2X mix, normalized to β-actin levels and analyzed using the comparative C_T_ method.

### Reporter gene assays

COS-7 cells were seeded in phenol red-free DMEM (Wisent) supplemented with 5% dextran-coated charcoal stripped serum (Sigma-Aldrich). Twenty-four hours after plating, cells were transfected with plasmid DNA using Fugene HD (Promega) according to the manufacturer’s recommendations. For the PPARγ transcriptional assays, cells were transfected with 10 ng of pRL-CMV (renilla; internal control), 25 ng of pcDNA mPPARγ, 25 ng of pCMV6 mRXR, and 125 ng of *aP2* enhancer-luciferase (aP2 enhancer-luc). Murine *aP2*-luciferase was a gift from Bruce Spiegelman (Addgene plasmid #8858). This 520 bp enhancer construct is 5.1 kb upstream from the transcriptional start site and contains a functional PPRE (PPARγ response element). Six hours after transfection, cells were treated with vehicle control and the indicated concentrations of TROG, FM550, TPP, IPTP, DPP, TBB, or TBPH. Twenty-four after treatment, cells were lysed using 1X Passive Lysis Buffer (Promega). Luciferase activity was quantified with the Dual Luciferase Assay kit (Promega) using the Glomax96 Luminometer (Promega). Luciferase activity was normalized to renilla levels and to vehicle control (DMSO).

### Chromatin immunoprecipitation (ChIP) assay

3T3-L1 preadipocytes were seeded on to 10 cm plates and grown to confluence. Two days post confluence cells were treated with differentiation media containing dexamethasone, troglitazone, 10 μM IPTP, or 20 μM TPP. Media was changed on days 2 and 4, and cells were harvested on day 6. Protein-DNA complexes were cross-linked using 1% formaldehyde for 10 minutes and the cross-linking was then quenched with the addition of 125 mM glycine for 5 minutes. Cells were then washed with PBS, harvested, and resuspended in lysis buffer (50 mM Tris-HCl [pH = 8.0], 150 mM NaCl, 1 mM EDTA, 1% Triton X-100, 0.1% Na-deoxycholate) containing protease inhibitors and sonicated 4 times for 10 minutes with a 30s on/off cycle. Soluble chromatin was collected by centrifugation and an aliquot of chromatin was removed for total input quantification. Supernatants were incubated with protein A agarose beads (Simga Aldrich; 50% slurry) for 1 hour at 4°C. The supernatant was then transferred to a new microcentrifuge tube and 2 μg of PPARγ antibody or rabbit IgG (sc-2027; Santa Cruz) was added. After overnight incubation, 30 μL of protein A agarose beads were added and incubated for 2 hours at 4°C. Beads were then washed as previously described [24], protein-DNA complexes eluted in 100 μL of elution buffer (TE, 1% SDS), and cross-links reversed by overnight incubation at 65°C. DNA was purified using a PCR purification kit (Biobasic, BS664) and eluted in 50 μL of TE. Recruitment of PPARγ to the *aP2* enhancer region was measured by qPCR using the following primer pair: 5`-TGCGACAAAGGCAGAAATGC-3`forward and 5`-GCTCTCTGGGTGGTGACTTC-3`reverse.

### Statistical analyses

All data were analyzed using a one-way analysis of variance followed by Tukey’s post-hoc test. For non-normal data, a Kruskal-Wallis one way ANOVA on ranks was performed, followed by Dunnett’s post-hoc test. Statistical analyses were performed using SigmaPlot 12.5 or GraphPad Prism software.

## Results

### Lipid accumulation after exposure to Firemaster^®^ 550 and its components in 3T3-L1 preadipocytes

**Fig 1:**
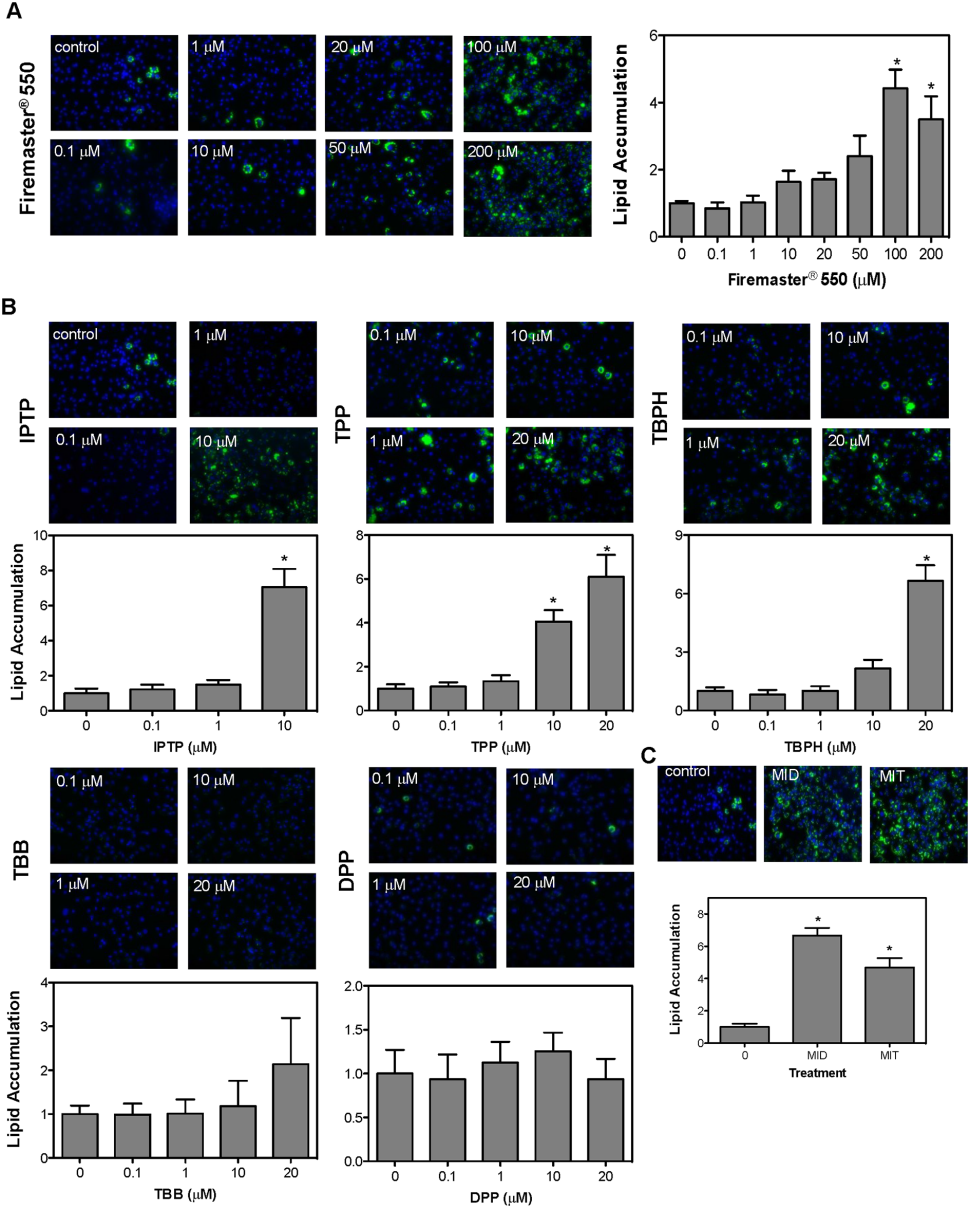
Lipid accumulation after exposure to Firemaster^®^ 550 and its components using 3T3-L1 preadipocytes. (**A**) Preadipocytes were differentiated as described in the Materials and Methods with vehicle with IBMX and insulin (MI) or increasing amounts of Firemaster^®^ 550 (0.1–200 μM) or with the FM550 components (**B**) IPTP, TPP, TBPH, TBB, and DBB (0.1–20 μM) and (**C**) 250 nM dexamethasone (MID), or 5 μM troglitazone (MIT). After 9 days lipid accumulation was visualized using Nile Red staining and then quantified. Lipid accumulation was normalized to DAPI staining and relative to vehicle-treated cells. The photographs are representative data obtained from experiments run in parallel with all the chemicals. Data represent mean ± SEM for *n* = 3–5 independent experiments performed in triplicate. Statistical significance *P<0.05 was determined relative to vehicle control (MI) using a one-way ANOVA followed by Tukey’s post-hoc analysis. Images were visualized using the Olympus IX71 fluorescent microscope at 200X magnification and are representative of at least three independent experiments.

To determine FM550 and its components’ adipogenic potential, lipid droplet formation was evaluated by Nile red staining which was subsequently quantified. Chemicals were non-cytotoxic, by microscopic examination and DAPI staining, except for 20 μM IPTP which was not used in our study (S1 Fig). Lipid staining was observed in cells exposed to increasing concentrations of FM550 (Fig 1A) resulting in a dose-dependent increase in lipid accumulation which was significantly higher than control after 100 μM and 200 μM FM550 treatment. However, increased lipid accumulation was seen at concentrations as low as 10 μM (~2-fold increase). The amount of lipid accumulation observed at the higher doses of FM550 was comparable to our positive controls dexamethasone and troglitazone (Fig 1C). The components of Firemaster^®^ 550 were then tested to determine the active chemicals responsible for FM550’s observed lipid accumulation (Fig 1B). The components comprised of IPTP, TPP, and TBPH all caused a significant increase in lipid accumulation (10 μM, 10 μM & 20 μM, and 20 μM respectively) with the level of lipid staining within the same range of our positive controls (Fig 1C). Treatment with increasing amounts of TBB or DPP, the metabolite of TPP, did not result in increased lipid accumulation suggesting that TBB or DPP are unlikely to contribute to the adipogenic effects observed for Firemaster^®^ 550. (Fig 1B).

### Protein expression of mature adipocyte markers following exposure to Firemaster^®^ 550 and its components

**Fig 2:**
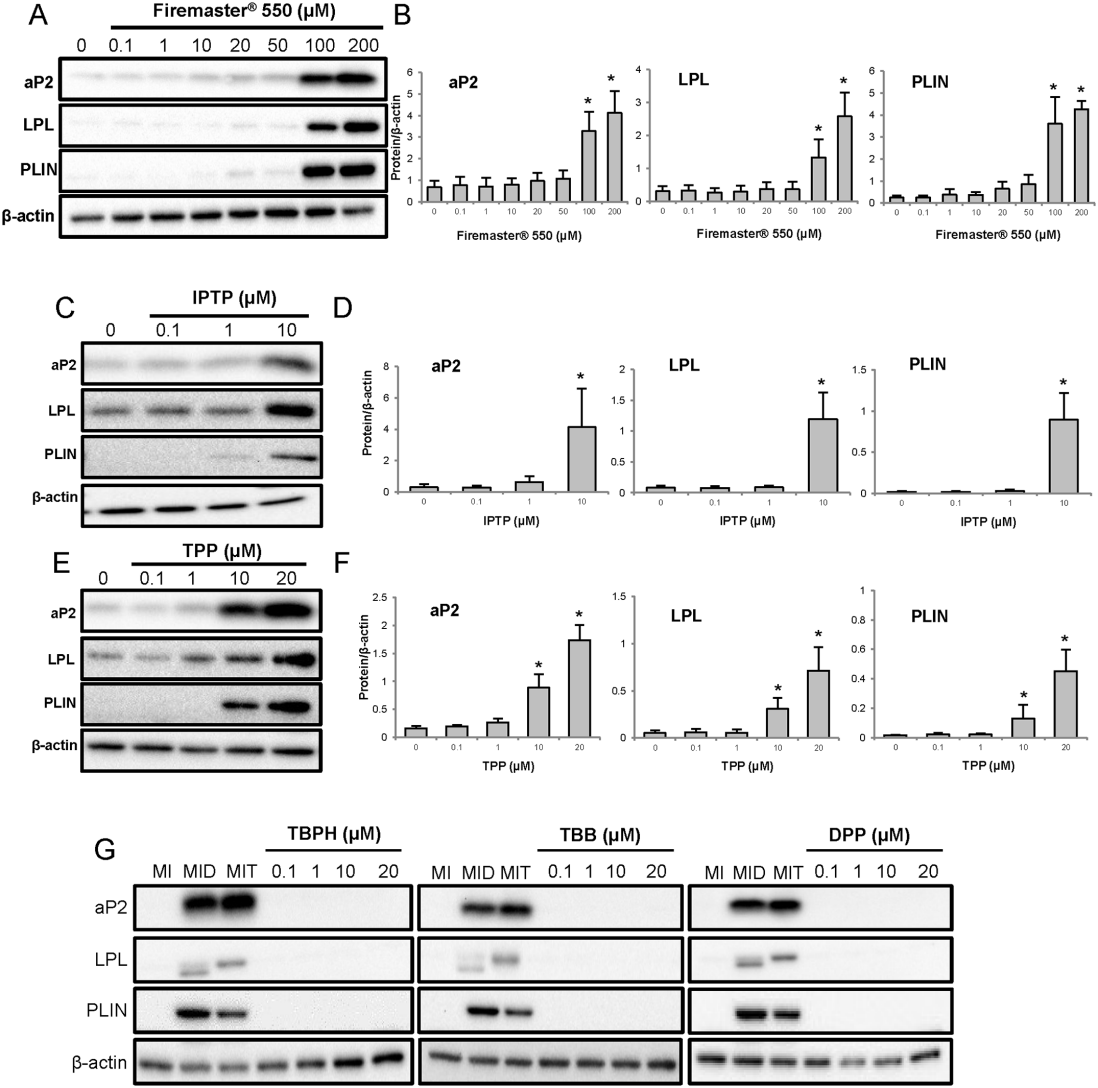
Protein expression in the mature adipocyte following exposure to Firemaster^®^ 550 and its components. Representative immunoblots depicting the ability of (**A**) Firemaster^®^ 550 (0.1–200 μM), and the components (**C**) IPTP (0.1–10 μM) and (**E**) TPP (0.1–20 μM) at inducing the expression of aP2, LPL, PLIN following 9 days of treatment. β-actin was used as the loading control. Quantification of aP2, LPL, and PLIN protein expression levels after (**B**) FM550 (**D**) IPTP (**F**) TPP treatment (*n* = 3–5) using ImageLab software (BioRad) and β-actin as loading control. (**G**) TBPH, TBB DPP treatments at day 9. The positive controls dexamethasone (MID) and troglitazone (MIT) are shown. Blots are representative of at least three independent experiments. *P<0.05 compared to MI control using a one-way ANOVA followed by Tukey’s post-hoc analysis. Data are expressed as mean ± SEM.

To further characterize the ability of FM550 and its components at inducing adipogenesis, proteins specifically expressed in the mature adipocyte (aP2, LPL, PLIN) were measured after nine days of chemical treatment. Exposure to increasing concentrations of Firemaster^®^ 550, resulted in a dose-dependent increase in aP2, LPL, PLIN expression (Fig 2A), with significant protein expression relative to control observed at the 100 μM and 200 μM exposure levels (Fig 2B), corresponding to the same doses we observed increased lipid accumulation. In accordance with the lipid accumulation results, IPTP and TPP treatment resulted in a dose-dependent increase in protein expression levels of aP2, LPL, and PLIN which was statistically significant at the higher doses (Fig 2C–2F). Interestingly, despite seeing enhanced lipid accumulation after TBPH exposure, no changes in protein expression of aP2, LPL, and PLIN were observed, consistent with previously reported results ([25], S2A Fig). Similar to our lipid quantification results, TBB and DPP treatment did not enhance the protein expression of aP2, LPL, or PLIN (S2B/S2C Fig). Our data show that FM550 and its components IPTP and TPP but not TBPH-induced lipid accumulation led to the expression of terminal differentiation markers. Furthermore, our data suggest that the components IPTP and TPP are responsible for FM550’s adipogenic effects.

### Temporal mRNA expression of transcription factors and terminal differentiation markers after exposure to Firemaster^®^ 550 and its components

**Fig 3:**
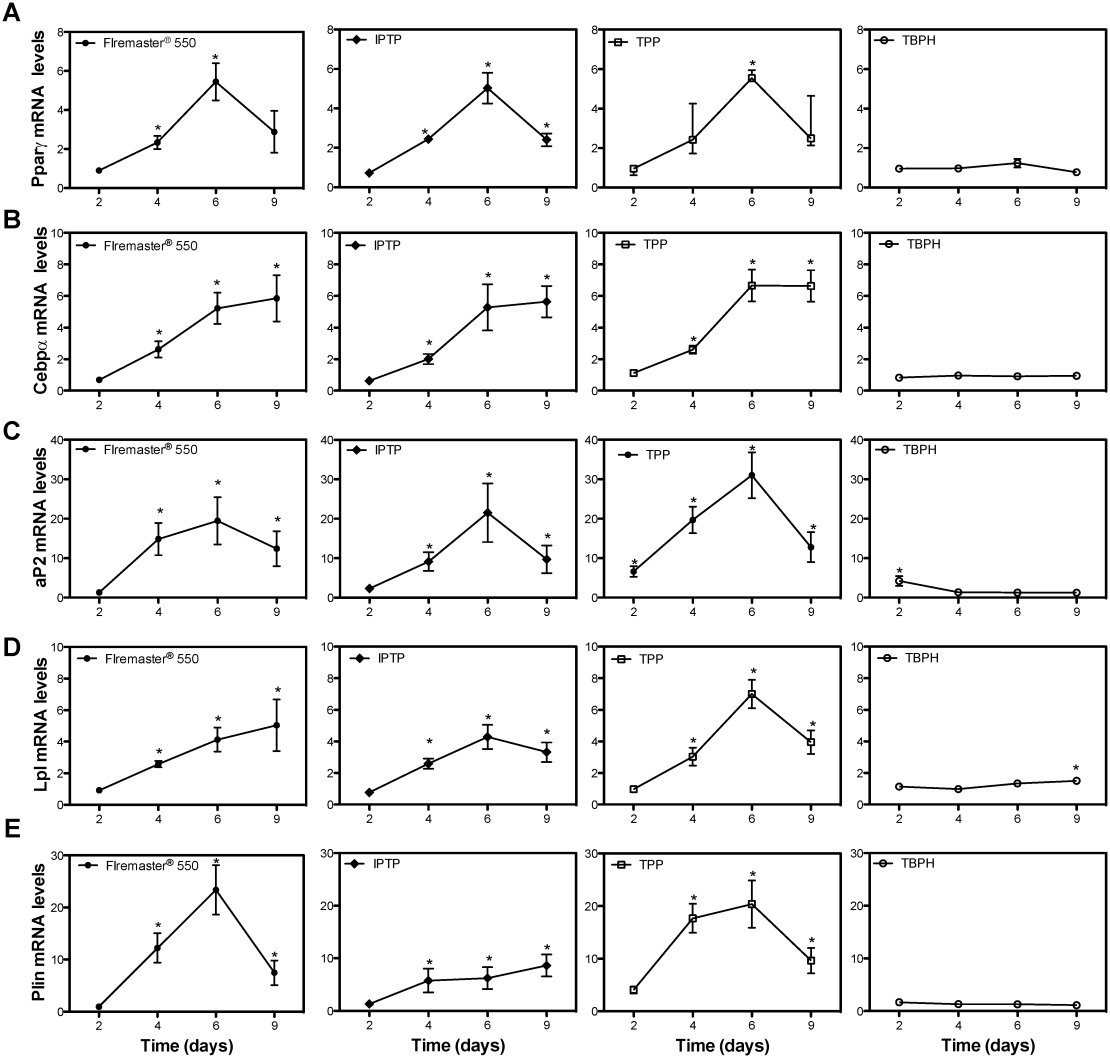
Temporal expression of transcription factors and terminal differentiation markers after exposure to Firemaster^®^ 550 and its components. mRNA expression levels were determined in 3T3-L1 preadipocytes 2, 4, 6, and 9 days post-treatment with the differentiation cocktail consisting of IBMX, insulin, and either 100 μM FM550, 10 μM IPTP, 20 μM TPP, or 20 μM TBPH. After the indicated time-points, RNA was extracted and reverse transcribed, and we measured the levels of (**A**) *Pparγ*, (**B**) *Cebpα*, (**C**) *aP2*, (**D**) *Lpl*, and (**E**) *Plin*, which were normalized to *β-actin* levels and relative to time-matched vehicle control (MI). Data represent the mean ± S.E.M (*n* = 3–5). * *P*<0.05 relative to time-matched vehicle control using a one-way ANOVA followed by Tukey’s post-hoc analysis.

To investigate the ability of FM550 and its components to activate the adipogenic process, 3T3-L1 cells were treated with the active chemicals and mRNA expression was determined throughout the differentiation process (days 2, 4, 6, 9). We tested 100 μM of FM550, 10 μM IPTP, 20 μM TPP, and 20 μM TBPH; all doses previously shown to cause significant lipid accumulation and/or protein expression of terminal differentiation markers. *Pparγ*, known as the master regulator of adipogenesis was significantly upregulated (~2.5-fold) after treatment with FM550 and IPTP on day 4 and its expression was maintained at later time points (Fig 3A). TPP treatment resulted in the significant upregulation of *Pparγ* on day 6 (6-fold). There was no significant upregulation of *Pparγ* despite seeing enhanced lipid accumulation on day 8 after TBPH treatment at all time points examined (Fig 3A). Similar to *Pparγ*, the expression of the transcription factor *Cebpα* was significantly upregulated on day 4 (2-fold increase) reaching maximal levels of expression on day 6 (6-fold increase) which was maintained until day 9 following FM550, IPTP, and TPP, but not TBPH treatment (Fig 3B). The expression of aP2, a protein primarily expressed in mature adipocytes was upregulated to a maximal 20-fold increase relative to control after FM550, IPTP, and TPP treatments (Fig 3C). Significant *aP2* upregulation was seen as early as day 2 (TPP treatment) but was significant on day 4 for FM550 and IPTP (Fig 3C). Interestingly, a small but significant upregulation of *aP2* was seen in cells exposed to TBPH in agreement with the observed lipid accumulation (Fig 3C). The expression of *Lpl* in 3T3-L1 exposed to FM550, IPTP, and TPP was significantly upregulated on day 4 and mRNA levels were sustained until day 9 (Fig 3D). Unlike its effect on aP2 which was seen early in the differentiation process, TBPH treatment resulted in a significant increase in *Lpl* expression on day 9, but to a much lower extent when compared to the other components (Fig 3D). The expression of *Plin* was significantly upregulated on day 4 and was sustained until day 9 after FM550, IPTP, and TPP treatment but not TBPH (Fig 3E). The level of *Plin* expression was similar for FM550 and TPP treatment while for IPTP treatment it was approximately 2-fold less. TBPH did not increase *Plin* expression at all time points examined (Fig 3E). Overall, our results suggest that FM550 and its components IPTP, TPP, but not TBPH upregulate the expression of key transcription factors and markers expressed in adipocytes throughout the differentiation process; confirming that IPTP and TPP play important roles in FM550’s adipogenic potential.

### FM550 and its components activate the PPRE-dependent aP2 reporter construct

**Fig 4:**
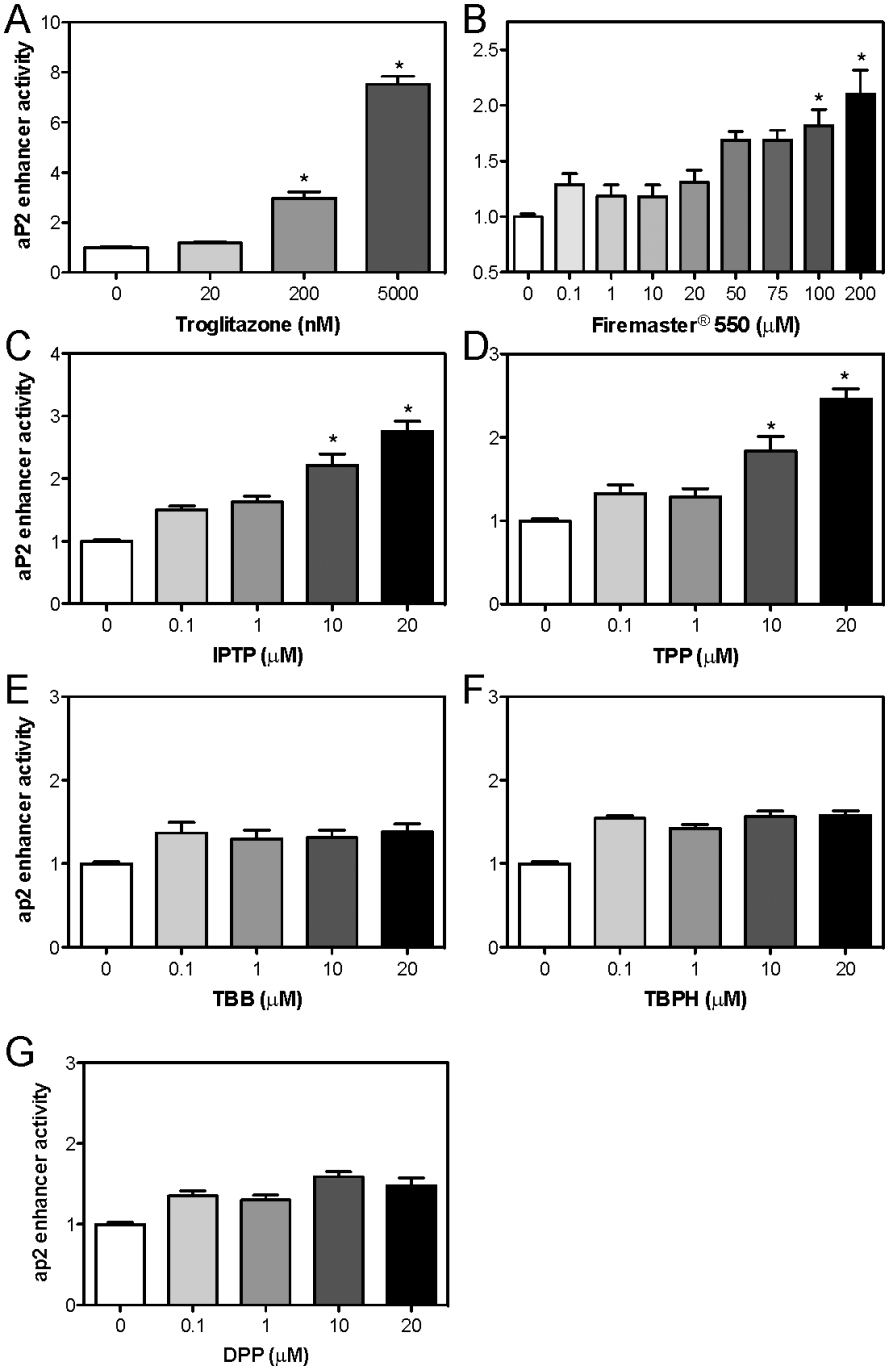
The ability of FM550 and its components to activate the PPRE-dependent *aP2* reporter construct which requires direct PPARγ activation. COS-7 cells were transfected as described in the Materials and Methods with pcDNA-mPPAR**γ**, pcDNA mRXR, *aP2 enhancer*-luciferase, and pCMV-RL and treated with increasing amounts of the positive control (**A**) Troglitazone (0.02–5 μM), (**B**) FM550 (0.1–200 μM), or the components (**C**) IPTP (0.1–20 μM) (**D**) TPP (0.1–20 μM) (**E**) TBB (0.1–20 μM) (**F**) TBPH (0.1–20 μM) (**G**) DPP (0.1–20 μM). Twenty-four hours after treatment reporter gene activity was determined. Data represent the mean ± S.E.M of three independent experiments. Significantly different (*P<0.05) reporter gene activity was analyzed relative to transfected vehicle-treated cells using a one-way ANOVA followed by Tukey’s post-hoc analysis.

Previous studies have shown that FM550 and the components IPTP and TPP can interact with PPARγ to activate a 3X PPRE-dependent luciferase system (17). It is unclear whether they can activate an endogenous PPRE-containing (PPARγ dependent) endogenous promoter. To address this, we investigated their ability to activate the *aP2* promoter using a 584-bp reporter construct containing an active PPRE located 5.4 kb away from the transcription start site (TSS) of *aP2 (*denoted *aP2* enhancer*)*. COS-7 cells were transfected with the 584-bp reporter construct, mPPARγ/mRXRα and PPRE-dependent transcriptional activity was determined. We first confirmed that the full PPARγ agonist troglitazone activates the *aP2* enhancer in a dose-dependent manner (Fig 4A). We observed a 3-fold increase after 200 nM TROG treatment which was further increased to 8-fold after 5 μM TROG treatment (Fig 4A). FM550 treatment resulted in small increases in *aP2* enhancer activity which was significant with 100 μM and 200 μM treatment. Interestingly the highest doses produced luciferase activity corresponding to 200 nM of TROG suggesting that FM550 is a weak agonist of PPARγ (Fig 4B). Similar experiments were completed in the presence of FM550’s components. In accordance to our mRNA expression data, the components IPTP and TPP caused a significant increase in luciferase activity after 10 μM and 20 μM treatment comparable to 200 nM troglitazone treatment (Fig 4C/4D). As expected, TBB, TBPH, and DPP did not enhance *aP2* luciferase activity confirming previous reports (Fig 4E–4G; (17)).

### TPP and IPTP-mediated aP2 expression but not lipid accumulation is inhibited by the selective PPARγ antagonist GW9662

**Fig 5:**
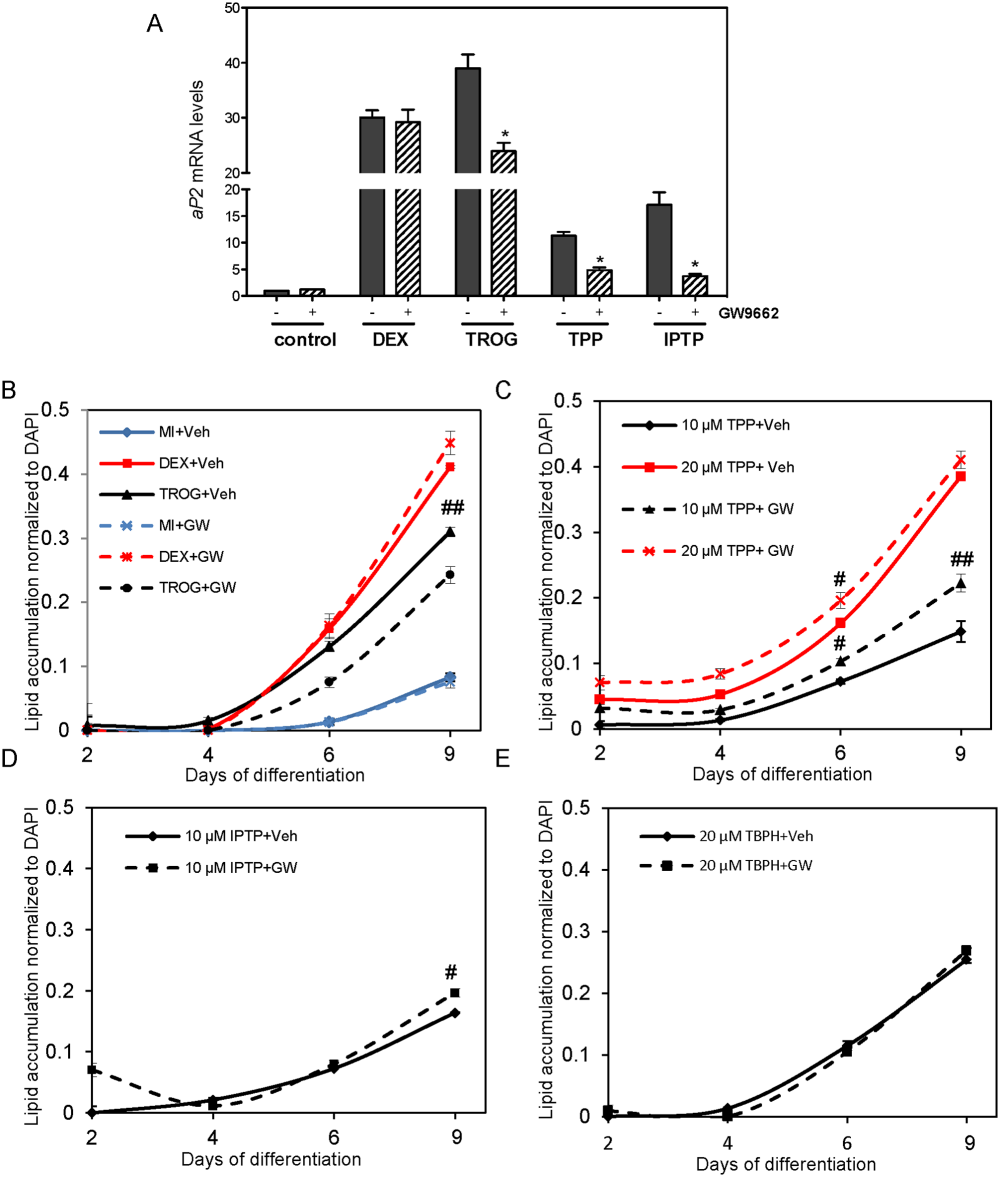
The effects of selective PPARγ antagonist GW9662 on aP2 mRNA expression and lipid accumulation in the presence of troglitazone, TPP, IPTP, and TBPH. Murine 3T3-L1 preadipocytes were induced to differentiate in the presence of 500 μM IBMX (M), 100 nM insulin (I), supplemented with indicated treatments: 5 μM troglitazone (TROG), 0.25 μM dexamethasone (DEX), 20 μM TPP, 10 μM IPTP, or 20 μM TBPH with either solvent control (Veh) or PPARγ inhibitor GW9662 (GW) twice daily. (**A**) At day 6 of differentiation mRNA expression levels of *aP2* were quantified by RT-qPCR normalized to MI control conditions. (**B-E**) At days 2, 4, 6, and 9 lipid accumulation was visualized using Nile Red staining and then quantified. Lipid accumulation was normalized to DAPI staining. Data represent mean ± SEM for n = 3 independent experiments. * denotes p<0.05 when comparing treatment to vehicle (MI) conditions (A). # denotes p<0.05, ## denotes p<0.01, and ### denotes p<0.001 when comparing to GW9662 at corresponding day of differentiation (B-E). Statistical analysis was performed by one-way ANOVA with Tukey’s post-hoc tests.

To further strengthen the role of direct PPARγ activation in TPP’s and IPTP’s adipogenic potential, we treated cells with the selective PPARγ antagonist GW9662 and measured the expression of aP2 mRNA via RT-PCR and lipid accumulation by Nile red. As expected, GW9662 caused a significant decrease in troglitazone-dependent but not dexamethasone-dependent *aP2* expression levels. *aP2* mRNA expression was also reduced in the TPP and IPTP treated cells co-treated with GW9662 (Fig 5A). GW9662 was also able to reduce the lipid accumulation of the troglitazone treated cells but not the dexamethasone treated 3T3-L1s (Fig 5B). Surprisingly, co-treatment of the cells with GW9662 and TPP, IPTP or TBPH did not result in a reduction in lipid accumulation (Fig 5C, 5D and 5E). Further, a slight enhancement in lipid accumulation was observed when cells were treated with TPP and IPTP in the presence of GW9662 (Fig 5C and 5D).

### The components IPTP and TPP induce recruitment of PPARγ to the aP2 enhancer region

**Fig 6:**
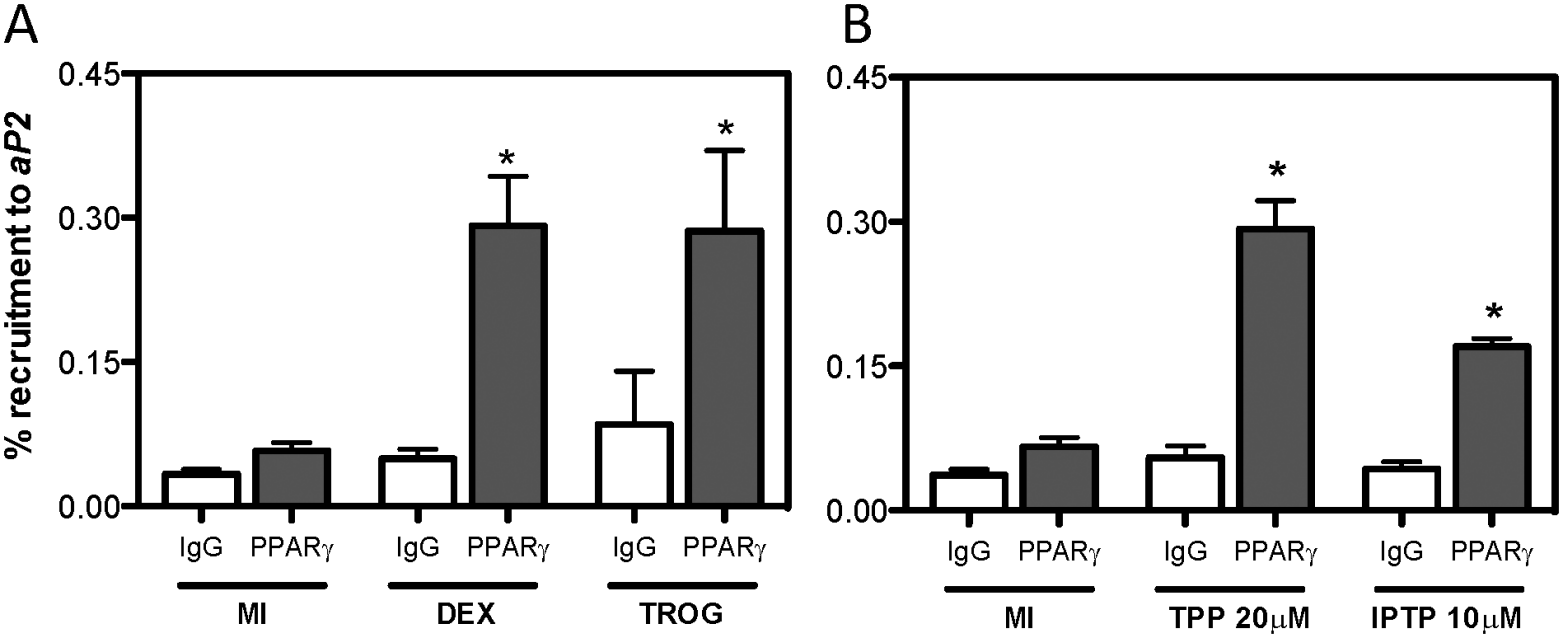
The components IPTP and TPP induce endogenous expression and recruitment of PPARγ to the *aP2* enhancer region. 3T3-L1 preadipocytes cells exposed to the differentiation cocktail for 6 days were used for the ChIP assay to measure the endogenous expression and recruitment of PPARγ to the *aP2 enhancer* region by RT-PCR. Positive controls dexamethasone (DEX) and troglitazone (TROG) were used and depicted in (**A**). (**B**) PPARγ recruitment to the *aP2 enhancer* region after exposure to 20 μM TPP and 10 μM IPTP was determined. Data represents the mean of *n* = 6 independent replicates ± SEM. Data were relative to 100% input DNA and significance was determined relative to control PPARγ recruitment using a one-way ANOVA followed by Tukey’s post-hoc analysis (*P<0.05).

To reinforce the results obtained for *aP2* expression levels and *aP2* enhancer luciferase activity, we investigated the recruitment of PPARγ to the endogenous PPRE 5.4 kb upstream from the TSS using the ChIP assay. The positive controls dexamethasone and troglitazone were also completed in conjunction with test chemicals to illustrate the importance of PPARγ in mediating their ability to induce aP2 expression. As PPARγ levels peaked on day 6 of differentiation (Fig 3A), this time point was selected to investigate the role of endogenous PPARγ in mediating TPP- and IPTP-dependent aP2 upregulation. Both dexamethasone and troglitazone increased the recruitment of PPARγ to the *aP2* enhancer region with comparable levels of recruitment for both compounds (0.3%, relative to 100% total input) (Fig 6A). Interestingly, treatment with the Firemaster^®^ 550 components TPP and IPTP caused significant recruitment of PPARγ to the *aP2* enhancer region, where 10 μM IPTP treatment resulted in about 0.2% recruitment and TPP resulted in 0.3% recruitment (Fig 6B). Taken together, our results illustrate that the Firemaster^®^ 550 components IPTP and TPP can induce endogenous PPARγ expression and its subsequent recruitment to the regulatory region of *aP2* leading to the observed increase in mRNA and protein expression levels.

## Discussion

In the United States, more than 35% of adults and nearly 17% of children (2–19 years) are obese [26]. The prevalence of obesity has become a worldwide epidemic affecting both developed and developing nations [27]. Though genetic predisposition and lifestyle choices are clear contributors to obesity, they cannot solely account for the rising obesity rates and global prevalence of the disease [28]. The obesogen hypothesis put forward by Blumberg and colleagues [29] suggests that exposure to EDCs can alter an individual’s susceptibility to metabolic disorders potentially accounting in part for the high obesity rates observed [28]. Identification of obesogenic compounds may be facilitated by the establishment of *in vitro* models such as the 3T3-L1 murine preadipocytes, and further investigations into the mechanism of action are also possible using this model. As previous studies have suggested FM550 and some of its components have the potential to act as obesogens by their ability to activate PPARγ, we set out to characterize the actions of FM550 and its components in the 3T3-L1s and determine its downstream effects, specifically by elucidating the effects of PPARγ induction on aP2.

Pillai *et al*. (2014) demonstrated in primary bone marrow cells that exposure to an osteogenic cocktail in the presence of FM550, IPTP, or TPP induces the expression of some adipogenic markers [30]. These results show that FM550 can promote adipogenesis at the expense of osteogenesis in bone marrow pluripotent cells; however, it was unclear whether these effects would be maintained using committed preadipocytes, which is suggestive of effects on residing preadipocytes in the adipose tissue. Our data show that exposure to FM550, TPP, and IPTP increased lipid accumulation, as well as the mRNA and protein levels of terminal differentiation markers in preadipocytes. However, the kinetics and levels of expression of the adipogenic markers differed between TPP and IPTP. For example, TPP and IPTP were able to activate PPARγ on the aP2 enhancer to similar levels and at similar concentrations (Fig 4). In fact, IPTP was slightly better at inducing PPAR-mediated transcriptional activity than TPP. However, the expression of specific genes in response to the two chemicals was different. While *aP2*, *Pparγ*, and *C/ebpα* expression levels were similar between IPTP and TPP, *Lpl* and *Plin* levels were higher in the TPP treated compared to the IPTP treated cells (~30 fold and ~6 fold compare to MI control respectively). While *aP2*, *Pparγ*, and *C/ebpα* are known targets of PPARγ, *Lpl* and *Plin* are genes that can be regulated by other transcription factors such as sterol regulatory element-binding protein (SREBP) 1 and liver X receptor (LXR) [31,32].

Interestingly, TBPH increased lipid accumulation but was only able to marginally induce the expression of adipogenic markers While these findings are perplexing, a recent study by Springer et al. (2012) showed similar results where TBPH induced lipid accumulation, but did not alter the expression of aP2 [25]. Furthermore, we show that TBPH, TPP and IPTP lipid accumulation was not reduced in the presence of the PPARγ antagonist GW9662. This shows that the regulation of lipogenesis is distinct from aP2 transcriptional regulation. It is still unclear what the mechanism by which TBPH TPP and IPTP induce lipid accumulation, however, it appears that it is not mediated via direct PPARγ activation. Structurally, the tri-aryl phosphates, which include TPP and IPTP, are similar to organotins which are strong activators of PPARγ [16,17]. For organotins, this tri-substitution is responsible for their interaction with PPARγ and hence their adipogenic potential. This structural composition is most likely also responsible for TPP and IPTP interactions with PPARγ and are likely the interaction sites with the ligand binding pocket of the receptor [29,30]. It has been previously determined that the brominated metabolite of TBPH, TBMEHP (mono-(2-ethylheyl) tetrabromophthalate), can activate PPARγ activity *in vitro* [25]. However, it has also been determined that this metabolite does not readily occur *in vitro* [33], consistent with our data showing that TBPH did not activate PPARγ transcriptional activity *in vitro*, or increased the expression of PPARγ target genes.

The differentiation transcriptional cascade in the 3T3-L1 preadipocytes is well-established with known early and late markers [8]. Our results show that FM550, IPTP, and TPP increased *Ppar*γ and *Cebpα* expression levels which are known to regulate each other’s expression in a positive feedback loop [34]. In addition, we show that FM550, IPTP, and TPP induced the expression of *aP2*. Although previous work using a 3X-PPRE-TK-luc luciferase reporter plasmid found that PPARγ activity was increased following treatment with FM550 components, it was unclear whether PPARγ activation in response to FM550 would also be true for an endogenous promoter. Using the endogenous regulatory region of *aP2*, a mature adipocyte marker and a direct target of PPARγ [35] we found that FM550, IPTP, and TPP significantly increased aP2 enhancer luciferase activity. AP2 is involved in fatty acid uptake and transport [12,36]. This is the first study to show that FM550 components IPTP and TPP enable PPARγ on an endogenous promoter. Further, using chromatin immunoprecipitation we show that IPTP and TPP treatments were able to enhance the recruitment of PPARγ to the endogenous *aP2* regulatory region used in our transcriptional reporter assays. Similar results were shown for MEHP where recruitment of transcriptional co-activators on endogenous PPARγ responsive promoters was observed after treatment of 3T3-L1 cells [37]. Future studies will establish if the recruitment of transcriptional co-activators are also recruited to the aP2 promoter in response to IPTP and TPP. Furthermore, our data show that IPTP- and TPP-induced *aP2* expression is mediated through direct PPARγ activation since the expression of *aP2* was inhibited by the PPARγ antagonist GW9662. The functional implications of aP2 upregulation are of particular importance, as its expression is highly correlated with metabolic disorders [38,39].

As we show that FM550 and its components TPP and IPTP induce adipogenesis using an *in vitro* model, it is important to consider the potential implications or limitations our findings have on human health. As FM550 is composed of 4 components that are also used individually in other applications, exposure resulting directly from use of the FM550 may be difficult to assess. Regardless, exposure to flame retardants occurs primarily through ingestion and inhalation of house dust, and studies have shown that TPP, TBB, and TBPH are readily detected in house dust samples [22,40–42]. Estimations of human exposure have relied on measurements of metabolites in urine samples. Hoffman *et al*. showed that the primary metabolite of TBB was detected in 72.4% of urine samples collected, suggesting that exposure to TBB is widespread [43]. Moreover, a recent study detected TBB and TBPH in all human hair and nail samples collected [44]. Interestingly, Mendelsohn *et al*. measured DPP, the primary urinary metabolite of TPP, in urine samples collected from women after application of nail polish and found that DPP levels increased 7-fold 10–14 hours following nail polish use [18]. These findings indicate that human exposure to the components of FM550, whether from the mixture or from other sources is likely ubiquitous and further studies regarding body burden measurements of these chemicals are required to assess their impact on metabolism, obesity and metabolism.

In conclusion, we demonstrate that FM550 and its components IPTP and TPP increase adipogenesis of 3T3-L1 preadipocytes. This increase is likely mediated by direct transactivation of *Pparγ*, although other modes of action cannot be ruled out. Further we show that the effect of IPTP and TPP on specific genes is different, indicating that these two components may have additional targets. Using the enhancer region of the endogenous *aP2* promoter region containing PPREs, we show that IPTP and TPP were able to recruit endogenous *Pparγ* to its target gene *aP2*, showing that the identified PPREs are functional and responsive to the chemicals. Further we show that the expression of *aP2* in response to IPTP and TPP was abolished in the presence of the Ppar*γ* inhibitor GW9662 indicating that Pparγ is required for FM550 components upregulation of the *aP2* transcripts. Further studies are required to assess the effects of FM550 components on human health, and to determine whether these flame retardants are any safer than the polybrominated flame retardants which they replaced.

## Supporting information

S1 Fig Preadipocytes were treated with vehicle with IBMX and insulin (MI) or (A) 250 nM dexamethasone (MID), or 5 μM troglitazone (MIT) or (B) increasing amounts of Firemaster^®^ 550 (0.1–200 μM) or with the FM550 components (C) TPP (D) IPTP (0.1–20 μM) as described in the Materials and Methods. After 9 days cytotoxicity was assessed by DAPI staining. Data represent mean ± SEM for n = 3–5 independent experiments performed in triplicate.
(TIFF)

S2 Fig Protein expression in the mature adipocyte following exposure to TBPH, TBB, or DPP. Representative immunoblots for aP2, LPL, and PLIN in response to treatment with (**A**) TBPH, (**B**) TBB (**C**) DPP for 9 days. In addition, the positive controls dexamethasone (MID) and troglitazone (MIT) are shown. Blots are representative of at least three independent experiments and β-actin was used as the loading control.
(TIFF)

S3 Fig Temporal expression of transcription factors and terminal differentiation markers after exposure to troglitazone. mRNA expression levels were determined in 3T3-L1 preadipocytes 2, 4, 6, and 9 days post-treatment with the differentiation cocktail consisting of IBMX, insulin, and either 100 nM and either 0.2 or 5 μM troglitazone. After the indicated time-points, RNA was extracted and reverse transcribed, and we measured the levels of (A) *Ppar*γ (B) *Cebpα*, (C) *aP2*, (D) and Plin which were normalized to *β-actin* levels and relative to time-matched vehicle control (MI). Data represent the mean ± S.E.M (n = 4).
(TIFF)
