# Supplementary figures and images for: Firemaster® 550 and its components isopropylated triphenyl phosphate and triphenyl phosphate enhance adipogenesis and transcriptional activity of peroxisome proliferator activated receptor (Pparγ) on the adipocyte protein 2 (aP2) promoter
Source: PLoS One. 2017 Apr 24;12(4):e0175855. doi: 10.1371/journal.pone.0175855 (PMC5402942; doi:10.1371/journal.pone.0175855)

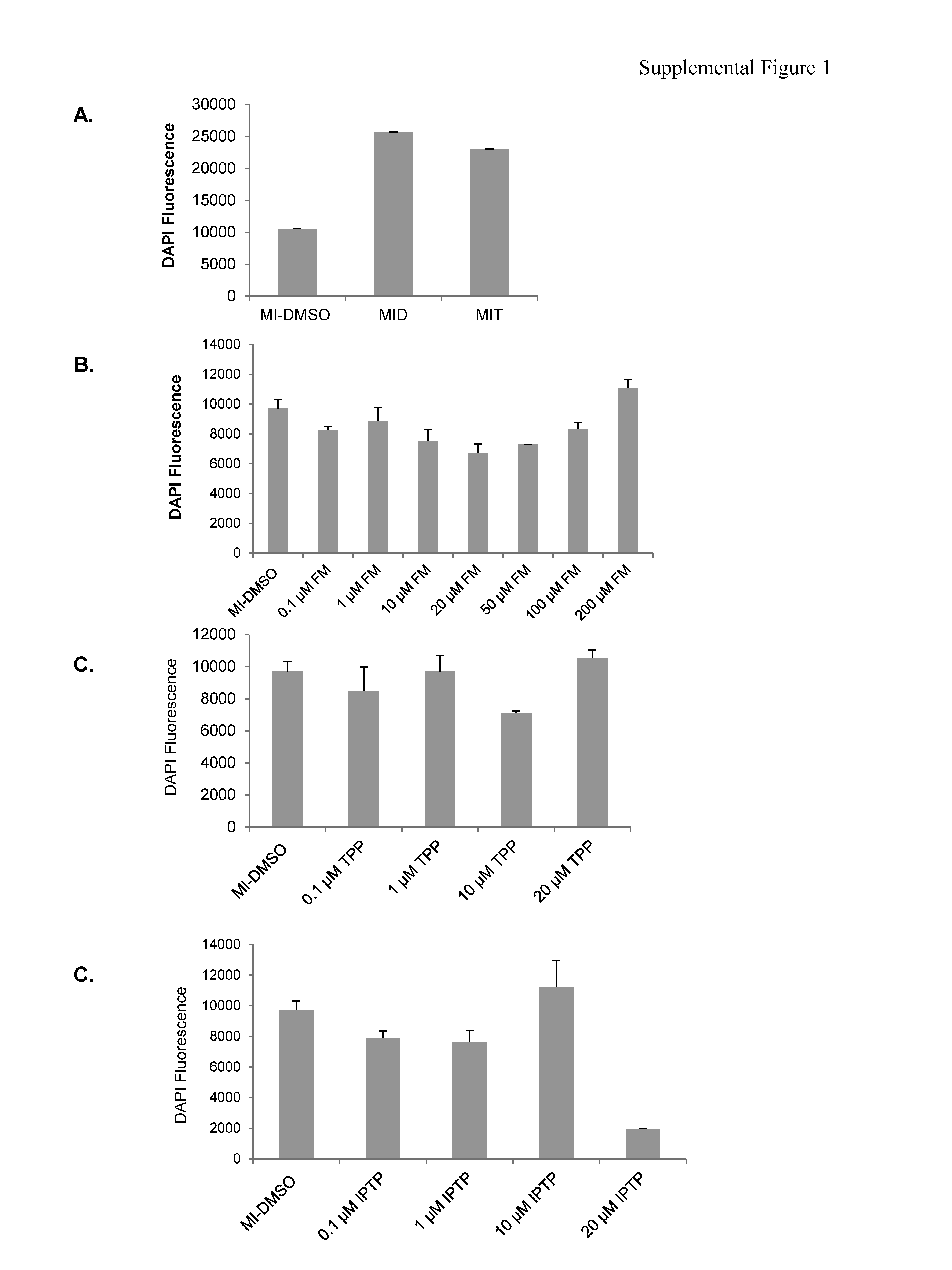

Supplement: S1 Fig — After 9 days cytotoxicity was assessed by DAPI staining. Data represent mean ± SEM for n = 3–5 independent experiments performed in triplicate. (TIFF) [file pone.0175855.s001.tiff]

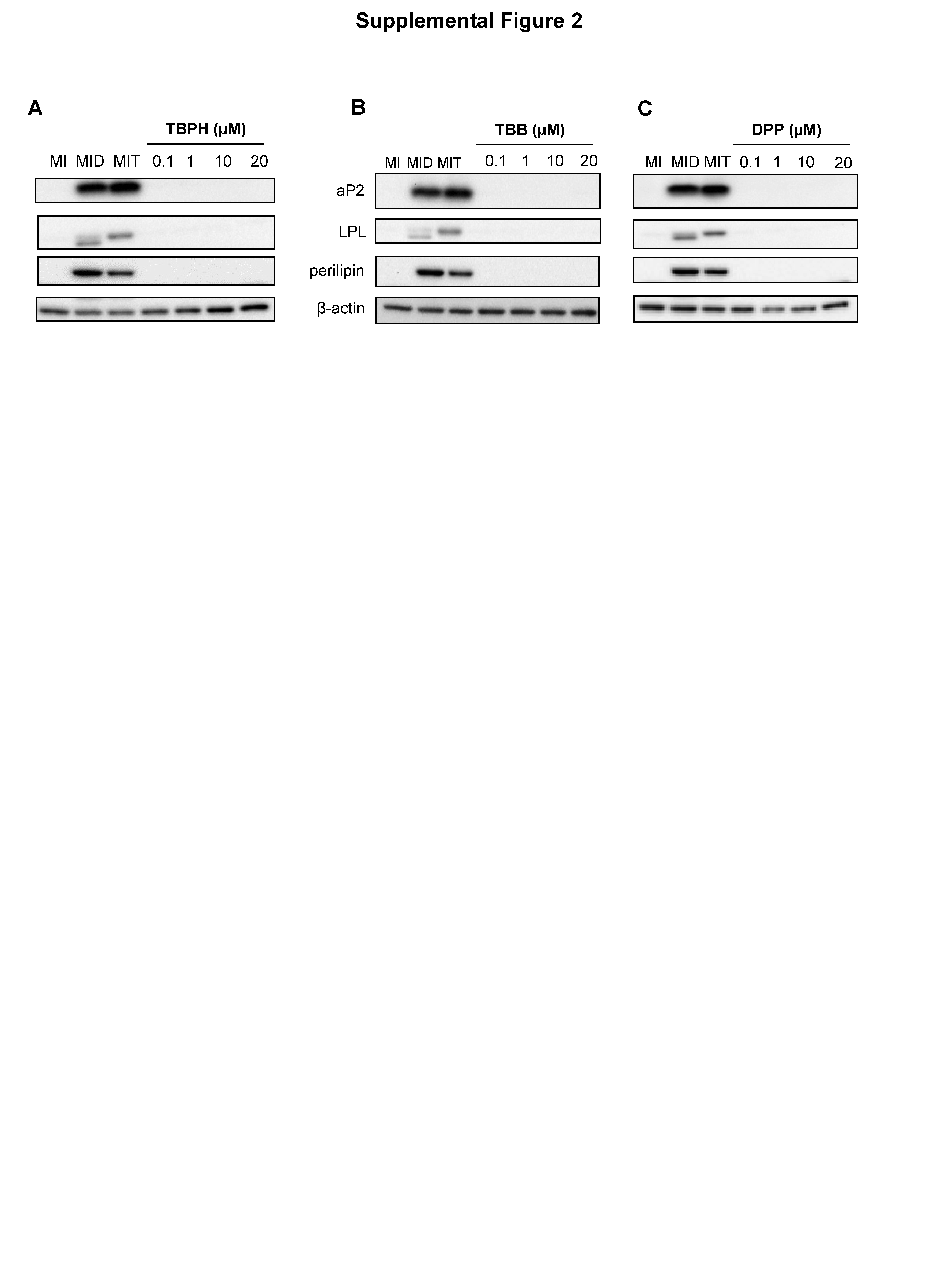

Supplement: S2 Fig — Representative immunoblots for aP2, LPL, and PLIN in response to treatment with (A) TBPH, (B) TBB (C) DPP for 9 days. In addition, the positive controls dexamethasone (MID) and troglitazone (MIT) are shown. Blots are representative of at least three independent experiments and β-actin was used as the loading control. (TIFF) [file pone.0175855.s002.tiff]

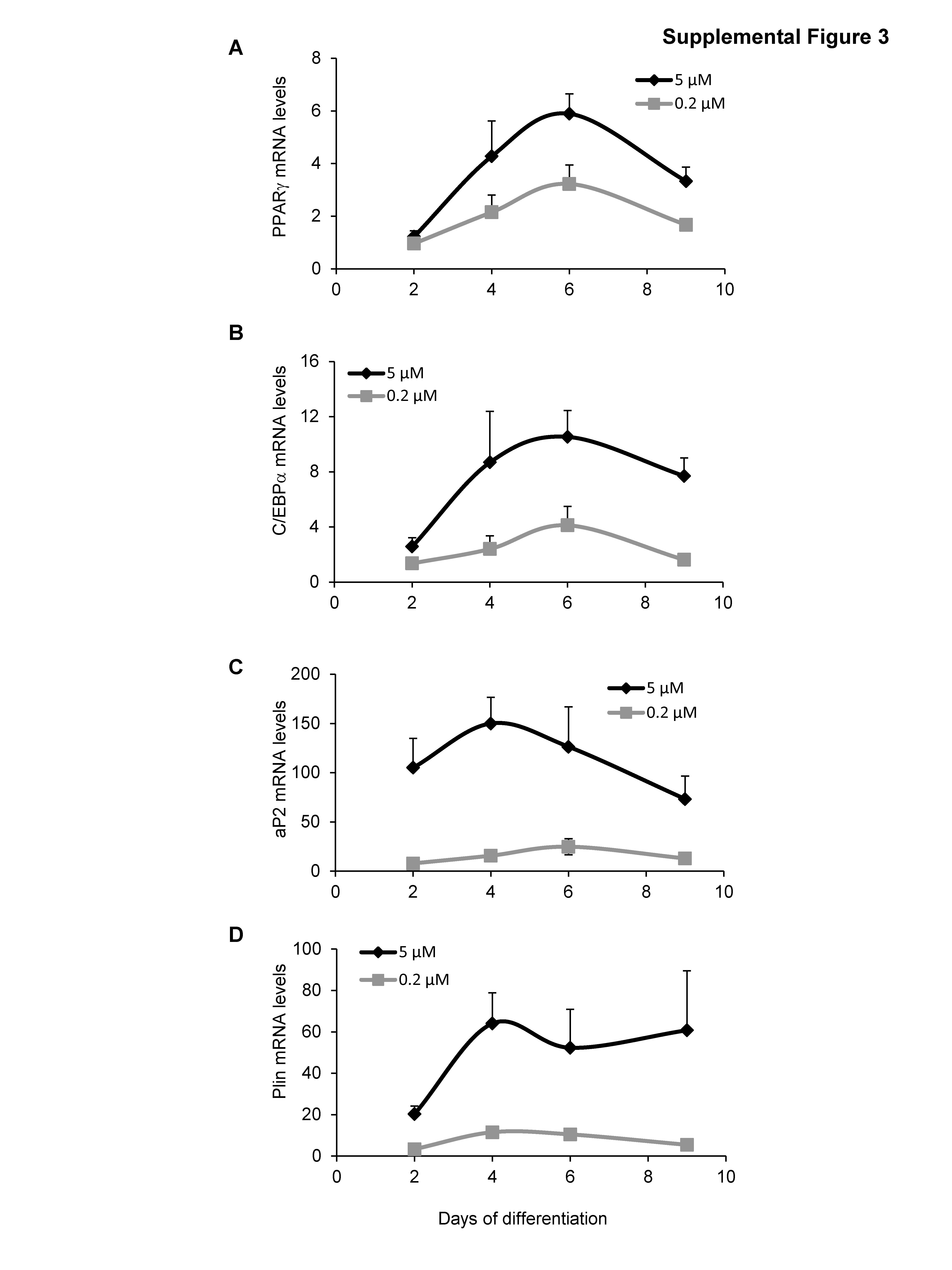

Supplement: S3 Fig — mRNA expression levels were determined in 3T3-L1 preadipocytes 2, 4, 6, and 9 days post-treatment with the differentiation cocktail consisting of IBMX, insulin, and either 100 nM and either 0.2 or 5 μM troglitazone. After the indicated time-points, RNA was extracted and reverse transcribed, and we measured the levels of (A) Pparγ (B) Cebpα, (C) aP2, (D) and Plin which were normalized to β-actin levels and relative to time-matched vehicle control (MI). Data represent the mean ± S.E.M (n = 4). (TIFF) [file pone.0175855.s003.tiff]
